# Self-Reported Physical Activity Behavior and Practice of Healthcare Professionals During the Second and Third Trimester of Pregnancy in Greece

**DOI:** 10.3390/clinpract15030045

**Published:** 2025-02-24

**Authors:** Vasileios Daglas, Nikolaos Kostopoulos, Michalis Mitrotasios, Antigoni Sarantaki, Maria Iliadou, Athanasios Moustogiannis, Maria Dagla, Evangelia Antoniou

**Affiliations:** 1 Laboratory of Midwifery Care During Antenatal and Post Natal Period-Breastfeeding, Department of Midwifery, School of Health & Care Sciences, University of West Attica, 12243 Athens, Greece; vdaglas@uniwa.gr (V.D.); esarantaki@uniwa.gr (A.S.); miliad@uniwa.gr (M.I.); lilanton@uniwa.gr (E.A.); 2 School of Physical Education and Sport Science, National and Kapodistrian University of Athens, 17237 Athens, Greece; nikkosto@phed.uoa.gr (N.K.); micmit@phed.uoa.gr (M.M.); 3 Department of Physiology, Medical School, National and Kapodistrian University of Athens, 17237 Athens, Greece; amoustogiannis@nyc.gr

**Keywords:** physical activity, pregnancy, behavior, obstetrician, midwife, cross-sectional study, second trimester, third trimester

## Abstract

**Background/Objectives:** The primary aim of the study was to investigate the self-reported behavior and practice of healthcare professionals (midwives and obstetricians) regarding physical activity during the second and third trimesters of pregnancy. The secondary goal of the study was to highlight sociodemographic and professional characteristics affecting the aforementioned behavior. **Methods**: The study was of cross-sectional design and was conducted between January 2022 and March 2023 with the participation of 235 midwives and obstetricians working in public and private facilities in the region of Attica, Greece. The participants completed a demographic characteristics form as well as a questionnaire structured to serve the aim of the study. Eight independent models of multivariate analyses of variance were performed. **Results**: Among the participants, over 97% recommended exercising during the second and third trimesters of pregnancy. Cardio exercises were the most commonly suggested type (93.1% in the second trimester and 88.5% in the third trimester), followed by relaxation exercises (87.7% and 89.8%, respectively). The majority recommended exercising 2–3 times per week, with session durations ranging from 15 to 30 min in the second trimester and 30 to 45 min in the third trimester. The intention for recommending physical activity during the second trimester was associated with the profession (*p* < 0.001), the personal attitude toward the necessity of exercising (*p* = 0.006), the participants’ belief regarding the importance of being informed about relevant recommendations (*p* = 0.003), and the degree of knowledge regarding the relevant international guidelines (*p* = 0.031). With respect to the third trimester, the associated factors included gender (*p* = 0.011), the participant’s profession (*p* = 0.006), the degree of knowledge regarding the relevant international guidelines (*p* = 0.008), their positive attitude toward physical activity during pregnancy (*p* = 0.011), and the degree of knowledge regarding the relevant international guidelines (*p* = 0.008). **Conclusions**: The aforementioned factors should be taken into consideration when designing interventions for the promotion of physical activity during pregnancy. The structure of relevant instruments would facilitate the evaluation of health professionals’ behavior toward physical activity.

## 1. Introduction

Almost all medical fields consider physical activity to be an important strategy to prevent a large variety of health difficulties or reduce the negative outcome if a diagnosis is already established [1]. The relevant literature has shown that those who engage in systematic physical activity are at lower risk of premature morbidity and mortality and several chronic debilitating conditions, such as cardiovascular disease [2,3]. Furthermore, regular exercise has been associated with reduced stress levels and overall better physical and mental health [4,5,6]. The direct impact of exercising on mental health has been recently supported by You and colleagues, who found that regular physical activity could reduce stress levels as well as depressive symptomatology [7].

Physical activity, including cardio, resistance/muscle strengthening, or relaxation exercises, has been associated with reduced obesity and its related comorbidities for non-pregnant as well as pregnant samples [8]. Antenatal exercise, which refers to exercising during gestation, is considered an important aspect for the health promotion of both the mother and the fetus [9]. As far as the fetus is concerned, physical activity during pregnancy can aid its growth, development, and future cognitive function [10].

With respect to the mother, regular physical activity during pregnancy may most importantly reduce the probability of developing gestational diabetes. Regardless of pre-gestational BMI, excessive weight gain during pregnancy has been associated with several impediments, such as congenital anomalies for the fetus and heart disease for the mother [11]. In addition, regular physical activity may decrease the risk of preeclampsia [12] as well as postpartum depression [13].

National and international healthcare organizations suggest and openly promote physical activity as a beneficial approach to support pregnant women with a healthy background [14]. For example, regular, moderate-intensity physical activity during pregnancy has been recognized by the World Health Organization as a means to enhance the maternal psychological state as well as avoid complications related to the pregnancy [15]. Likewise, the guidelines published by the National Institute for Health and Care Excellence (NICE) highlight the benefits of antenatal exercise in promoting women’s health during pregnancy [16]. Similarly, the American College of Obstetricians and Gynecologists (ACOG) acknowledges that individualized suggestions for antenatal exercise can improve women’s cardiovascular and musculoskeletal health and overall quality of life during gestation [17].

Despite this global endorsement of physical activity by leading authorities of obstetrics, research has shown that women during pregnancy lack information regarding the benefits of physical activity during the gestational period [18]. When exploring the roots of this unawareness, sociodemographic factors such as the mother’s financial and cultural background were found [19]. The literature has shown that among the factors for this lack of knowledge is the low rate of relevant recommendations by healthcare professionals [20]. In relation to the latter, research has highlighted that the low rate of recommending physical activity to pregnant women occurs due to the limited training of healthcare professionals regarding the physical activity benefits during gestation [21,22].

Healthcare professionals, including midwives and obstetricians, constitute probably the main source of information and guidance during pregnancy. Their recommendations and attitudes toward physical activity may play an instrumental role in pregnant women’s willingness to engage in exercise [23]. Studies have shown that women who receive clear and supportive advice from their healthcare providers are more likely to adhere to physical activity guidelines, leading to improved maternal and fetal health outcomes [14]. Therefore, enhancing the training and awareness of healthcare professionals regarding the benefits of exercise during pregnancy is crucial for promoting healthy behaviors among expectant mothers. Despite the suggestions by international guidelines and the underlined role of midwives and obstetricians, the relevant updated research is scarce and limited to non-European populations [24,25,26], while only half of Greek healthcare professionals recommend exercise to pregnant women in the first trimester of pregnancy and the majority do not routinely recommend a specific frequency and duration of exercise [23]. The primary aim of this study was to investigate the self-reported behavior and practice of healthcare professionals (midwives and obstetricians) in Greece regarding physical activity recommendations during the second and third trimesters of pregnancy. The secondary objective was to examine the extent to which sociodemographic (e.g., age, gender) and professional characteristics (e.g., level of education, years of experience, type of workplace) influence these recommendations.

## 2. Materials and Methods

### 2.1. Study Design

The present was a cross-sectional study and was conducted between January 2022 and March 2023. The study was held among healthcare settings within the region of Attica, Greece, was consistent with the Declaration of Helsinki, and was approved by the committees of each setting in which it was conducted [Ref. Number (1st public hospital): 41/20-01-22, Ref. Number (2nd public hospital): 1480/28-01-22, and Ref. Number (private hospital): 9-12-2022] and by the 1st Regional Health Authority of Attica [Ref. Number: 21855/20-05-22].

### 2.2. Sample

The sample’s recruitment was performed by the primary researcher in person based on availability. Initially, 447 (281 midwives and 166 obstetricians) healthcare professionals were approached and informed in detail of the research’s aim. Of those, 235 (153 midwives and 84 obstetricians) agreed to participate and provided their signed consent. The participation response rate was 53%. No formal power analysis or sample size calculation was conducted. The study’s sample included healthcare professionals from both public and private healthcare facilities in Attica, Greece. Eligible participants were required to hold a midwifery or medical school diploma, possess a valid license to practice, and have at least one year of experience in monitoring pregnant women. Trainee obstetricians were not excluded from participation, whereas midwifery and medical students were ineligible. No additional exclusion criteria were applied. In total, 75.1% of the sample was composed of women, while the sample’s mean age was 40.6 years, 64.6% were midwives, and 35.4% were obstetricians. The mean years of working experience was 14.5 years, and 47.3% worked in the public sector.

### 2.3. Procedure

All eligible participants based on the inclusion/exclusion criteria were approached and invited by the primary researcher in person. Each participant was thoroughly informed about the aims of the study and could proceed to the relevant measurements only after providing their signed consent form. Following, the researcher coded each participation and entered the completed form into a pre-structured database.

### 2.4. Measurements

Participants had to complete a form regarding their sociodemographic and professional characteristics and a questionnaire structured to serve the aims of the study. More specifically, participants had to complete:

*Demographics and professional characteristics form*: Participants had to complete their age, gender, educational level, their specialty and professional experience in years, the setting they were employed at, and in the case of obstetricians, their position and experience in monitoring low-risk pregnant women. With respect to midwives, they had to complete whether they had experience in providing antenatal counseling to pregnant women or not and their years of experience in providing antenatal counseling to pregnant women.

*Self-report on behavior regarding recommendations for physical activity*: Participants had to complete a self-report questionnaire consisted of six items regarding their behavior and attitude toward physical activity during pregnancy. For four of the six items, responses were given on a binominal scale (yes/no), while for the remaining two (frequency and duration of suggested physical activity), responses were given on six- and five-point scales (not recommended, 15–30 min, 30–45 min, >1 h, no limit, and not recommended, 1 day/week, 2–3 days/week, 4–5 days/week, 5–6 days/week, everyday), respectively. The questionnaire was structured in the Greek language to serve the research purpose of the research team, while its completion by the participants was held in person.

### 2.5. Statistical Analyses

Descriptive statistics are presented in terms of absolute (*n*) and relative (%) frequencies for categorical variables, whereas for continuous variables, means and standard deviation were used. Fisher’s exact and *χ*^2^ tests were performed to determine possible nonrandom associations between categorical variables. To analyze the factors influencing healthcare professionals’ recommendations regarding physical activity, we conducted eight independent multivariate analyses of variance (MANOVA). Each model examined different aspects of physical activity recommendations, including the type of exercise suggested, frequency, and duration. The dependent variables in these models were the specific recommendations provided by participants, while the independent variables included participants’ demographic characteristics. Interaction effects were also examined to explore whether specific professional characteristics influenced the likelihood of recommending different types, frequencies, or durations of exercise. Data analyses were performed using the Statistical Package for Social Sciences version 22.0. Statistical significance (*p*) was set at 0.05.

### 2.6. Ethical Considerations

Eligible candidates were thoroughly informed about the study’s purposes and were recruited only after they provided their written consent. Anonymity was maintained by coding the responses. Access to the database of responses was available solely to the research team. No financial or other type of remuneration was provided to the participants and all retained the right to withdraw from the study at any timepoint until the statistical analyses began.

## 3. Results

### 3.1. Descriptive Statistics Regarding the Recommendations of Physical Activity During the Second and Third Trimesters of Pregnancy

The vast majority of midwives and obstetricians participating in the research claimed that they recommend exercise during pregnancy in the second (98.7%) and third trimesters (97.8%). Cardio exercises were the most common recommendation during the second trimester for 93.1% of the participants. The same type of exercise was recommended for the third trimester for 88.5% of the participants. A total of 54.9% of the participants recommended a frequency of 2–3 per week for the second trimester. With respect to the third trimester, 52% recommended the same frequency (2–3 times/week). With respect to the duration of activity during the second trimester the majority of participants (35.4%) were in favor of 15–30 min per exercise session, while for the third trimester, 32.9% recommended 30–45 min per session. The descriptive statistics of the recommendations are presented in Table 1.

### 3.2. Associations Between Occupational Characteristics and Recommendations Regarding the Type, Frequency, and Duration of Physical Activity During the Second and the Third Trimesters of Pregnancy

For the exploration of the relationship between occupational characteristics and recommendations regarding physical activity, the *χ*^2^ and Fisher’s exact tests were applied. The results showed that midwives with prenatal counseling experience were more likely to recommend cardio activity compared to those with no relevant experience (*χ*^2^ = 3.849, *p* = 0.050). With respect to the second trimester, resistance/strengthening exercises were more recommended by obstetricians compared to midwives (*χ*^2^ = 6.613, *p* = 0.010), health professionals with an MSc/PhD diploma compared to those without tertiary studies (*χ*^2^ = 4.457, *p* = 0.035), and health professionals of the public sector and primary health care in relation to the remaining participants (*χ*^2^ = 8.222, *p* = 0.042).When examining the fields of expertise (midwives and obstetricians), those who worked as freelance obstetricians were more likely to recommend this type of exercise compared to those in the public sector or private healthcare facilities (through the Fisher’s exact test) (*p* = 0.031), while midwives experienced in prenatal counseling were more likely to recommend this type of exercise compared to those with no relevant experience (*p* = 0.012). Details regarding the sample’s occupational characteristics and physical activity recommendations are presented in Table 2.

Regarding the third trimester, cardio physical activity was more likely to be recommended by female participants compared to males (*χ*^2^ = 6.067, *p* = 0.014) and midwives experienced in prenatal counseling compared to those with no relevant experience (*χ*^2^ = 17.504, *p* < 0.001). Regarding resistance/strengthening exercises, these were more likely to be recommended by midwives with experience in prenatal counseling compared to those with no relevant experience (*χ*^2^ = 8.823, *p* = 0.004). The associations between occupational and demographic characteristics and the type of physical activity are presented in Table 3.

With respect to the recommended frequency independently of the type, freelancers were more likely to recommend physical activity during the third trimester, with the recommendation ranging from one day/week to daily (*χ*^2^ = 16.056, *p* = 0.013), while regarding midwives, those experienced in prenatal counseling were more likely to recommend physical activity compared to those with no relevant experience, and this recommendation ranged between four days/week to daily exercising (*χ*^2^ = 16.056, *p* = 0.050). When examining the recommended frequency based on the type of exercise, statistically significant results were found only for resistance/strengthening exercises. More particularly, freelance midwives were more likely to recommend exercising (four days/week to daily) compared to those in other work sectors (*χ*^2^ = 6.720, *p* = 0.035). Regarding the duration of activity, male healthcare professionals were more likely to recommend a 15–30 min exercise compared to women, who recommended 30–45 min (*χ*^2^ = 15.283, *p* = 0.002), while obstetricians were more likely to recommend a 15–30 min exercise compared to midwives, who recommended 30–45 min (*χ*^2^ = 16.378, *p* = 0.001). Details of the occupational and demographic characteristics in relation to the frequency and duration of physical activity are presented in Table 4.

### 3.3. Examination of the Predictive Factors for the Recommendations for Physical Activity During the Second Trimester

In order to examine the predictive value of demographic and occupational factors on the recommendations for physical activity during the second trimester, three different and independent dichotomous logistic regression analyses were performed. The first model examined factors that could influence the recommendations for resistance/strengthening physical activity. With respect to profession, midwives were significantly less likely to recommend resistance/strengthening exercises during the second trimester compared to other professions (*p* < 0.001). When examining the role of their knowledge about the relevant guidelines, participants with least or moderate knowledge were significantly less likely to recommend these exercises (*p* = 0.003). The model explained 12.7% of the total variance (*R*^2^ = 0.127) of recommendations for resistance/strengthening exercises. The second model examined factors that could influence the recommendations for relaxation exercises. Participants who perceived moderate benefits were significantly less likely to recommend relaxation exercises (*p* = 0.006), while a high level of knowledge was associated with a higher likelihood of recommending them (*p* = 0.031). The model explained 14.6% of the total variance of recommendations for relaxation exercises (*R*^2^ = 0.146). The third model examined factors that could impact not specifying the frequency of activity. Freelance health professionals were less likely to specify the frequency of activity (*p* = 0.031), while a strong belief in the benefits of activity was associated with a lower likelihood of not specifying frequency (*p* = 0.017). The model explained 9% of the total variance, with the *R*^2^ being equal to 0.092.

### 3.4. Examination of the Predictive Factors for the Recommendations for Physical Activity During the Third Trimester

In order to examine the predictive value of demographic and occupational factors on the recommendations for physical activity during the third trimester, five different and independent dichotomous logistic regression analyses were performed. The first model examined factors that could influence the recommendations for cardio physical activity. The results showed that women were significantly more likely to recommend cardio exercises during the third trimester (*p* = 0.011), while participants who perceived moderate benefits were significantly less likely to recommend them (*p* = 0.011). The model explained 10.9% of the total variance of the recommendations for cardio physical activity (*R*^2^ = 0.109).

The second model examined factors that could influence the recommendations for resistance/strengthening activity. Midwives were significantly less likely to recommend this type of exercise during the third trimester (*p* = 0.006), whereas a higher level of knowledge of the relevant international guidelines was associated with a higher likelihood of recommending them (*p* = 0.008). In addition, it was found that those who noted the least/moderate necessity for information on the topic were significantly less likely to recommend resistance/strengthening activity (*p* = 0.007). The model explained 12.9% of the total variance of the dependent variable (*R*^2^ = 0.129).

The third model examined factors that could affect the possibility of suggesting a specific frequency of physical activity. The analysis highlighted that the freelance health professionals were less likely to suggest a frequency (*p* = 0.002) compared to those working in primary healthcare (*p* = 0.009). The model explained 14.2% of the total variance of the dependent variable (*R*^2^ = 0.142). The fourth model explored factors that could influence the recommendations for exercise sessions of 15 to 30 min. The results showed that midwives were significantly less likely to recommend this duration (*p* < 0.001), while believing that exercise is associated with small complications increased the likelihood of recommending this duration (*p* = 0.012). In addition, participants who noted the least necessity for information were less likely to recommend 15–30 min of exercise (*p* = 0.012). The model explained 16.1% of the total variance of the dependent variable (*R*^2^ = 0.161). The fifth model was also taken under consideration, and the recommendation for 30–45 min exercise sessions was set as the dependent variable. The results revealed that older age was significantly associated with a lower likelihood of recommending this duration (*p* = 0.003), with the model explaining 6.1% of the total variance.

## 4. Discussion

Pregnancy constitutes one of the most important periods of life for a woman. Being physically active during this period is one of the most suggested strategies to support the mental and physical wellbeing of healthy women. The aim of the present study was to investigate the behavior and practice of midwives and obstetricians of Attica regarding physical activity during the second and third trimesters of pregnancy and, simultaneously, detect any possible occupational or/and sociodemographic factors that could influence this behavior.

The results of this study highlighted the high frequency of recommendations for physical activity by midwives and obstetricians in the region of Attica, Greece. The findings indicate that the majority of midwives and obstetricians in Greece recommend physical activity during the second and third trimesters, with cardio exercises being the most frequently suggested, followed by relaxation and resistance/strengthening exercises.

These recommendations align with international guidelines, which emphasize the importance of a balanced exercise regimen during pregnancy. This practice aligns with international guidelines that support moderate-intensity physical activity to promote the health and wellbeing of both the mother and fetus [27]. The World Health Organization (WHO) advises that pregnant women engage in at least 150 min of moderate-intensity aerobic activity per week, incorporating muscle-strengthening exercises at least twice a week to enhance overall health and reduce pregnancy-related complications. Similarly, the American College of Obstetricians and Gynecologists (ACOG) supports a combination of aerobic and resistance training exercises to improve cardiovascular health, maintain muscle tone, and reduce the risk of gestational diabetes and excessive weight gain. The National Institute for Health and Care Excellence (NICE) also promotes moderate-intensity physical activity throughout pregnancy, with a particular focus on individualized exercise recommendations tailored to the woman’s health status and previous activity levels. Additionally, this finding aligns with previous research suggesting that the majority of midwives and obstetricians include physical activity in their recommendations for a healthier gestation [25,28,29,30].

The present study confirms that the recommendations provided by Greek healthcare professionals largely reflect these international guidelines. Nevertheless, the findings were not universal; some variations in the recommendations were noted, and these were related to various socio-demographic and professional characteristics of healthcare professionals that are discussed below. The results showed that practitioners with higher educational levels (MSc and PhD diplomas) were more likely to suggest regular exercising during the second and third trimesters of pregnancy. This may be attributed to the fact that practitioners with higher education have greater exposure to current research and evidence-based practices, as well as more specialized knowledge about the benefits of physical activity [31]. In addition, graduate and doctoral programs often encourage critical thinking and an analytical approach [32], which may help obstetricians and midwives to better appreciate the importance of exercise during pregnancy.

However, the variations in the frequency and duration of exercise suggested by different professionals underscore the need for more standardized education and training on antenatal physical activity. Enhancing healthcare professionals’ familiarity with specific recommendations from the WHO, ACOG, and NICE may improve the consistency and accuracy of exercise prescriptions for pregnant women, ultimately leading to better maternal and fetal health outcomes.

Another finding of this study showed that midwives experienced in prenatal counselling were more likely to suggest physical activity either during the second or the third trimester compared to those with no relevant experience. A plausible explanation for this outcome could be the fact that previous experience and training in the needs and concerns of pregnant women may allow midwives to feel more comfortable and confident in recommending physical activity. In addition, this finding aligned with similar research, which highlighted that midwives who are experienced in counseling were more likely to suggest physical activity as they felt more confident in answering all queries and doubts of pregnant women [21]. Their experience helps them to better understand the benefits of exercise for maternal and fetal health and offer more informed and safe advice to pregnant women [33]. In addition, positive attitudes toward physical activity were associated with increased frequency of recommendations.

These findings highlight the need for continued education and awareness of healthcare professionals about current guidelines and the benefits of physical activity during pregnancy. Improving knowledge and understanding of these issues can lead to more consistent and evidence-based recommendations, helping to promote the health of pregnant women and their fetuses.

Another factor that was noted as influencing the recommendation of physical activity was gender. The results showed that female health professionals were more likely to recommend exercise during the third trimester of pregnancy. This difference can be attributed to many factors, such as female professionals’ greater empathy and understanding of pregnant women [34], and perhaps their personal experience. In addition, female healthcare professionals may be more aware and knowledgeable about the benefits of physical activity during pregnancy, which is reinforced by their professional and personal experience [35]. Previous studies have shown that female health professionals are often more likely to adopt preventive and holistic approaches to healthcare [36,37], which could include promoting physical activity.

With respect to the duration and frequency of exercising, some factors affected the recommendations of the sample as well. Male practitioners may be more conservative in their recommendations, fearing potential complications, and therefore recommend shorter exercise durations. This finding may be related to the different educational approach and priorities set when training obstetricians compared to midwives. In addition, the lack of extensive education about physical activity and its benefits during pregnancy may constitute them reluctant to recommend longer durations of physical activity. Previous studies have shown that increased knowledge and education about the benefits of physical activity can lead to more ambitious recommendations [38].

Furthermore, the study revealed that health professionals who are aware of established guidelines are more likely to recommend specific duration and frequency of exercise to pregnant women. This could suggest that education and information about the benefits and safe practices of exercise during pregnancy are crucial to providing accurate and adequate recommendations. Research has shown that health professionals with a good understanding of international guidelines tend to encourage the endorsement of a healthier lifestyle [39], recognizing its numerous benefits for maternal and fetal health. In addition, older practitioners and those with more experience in prenatal counseling seem to recommend more frequent and more intense exercise. These findings highlight the importance of continuing to educate and inform healthcare professionals so they can provide the most informed and safe recommendations for physical activity during pregnancy.

### Strengths and Limitations of the Study

This study has several strong points that need to be noted. First, the large sample of healthcare professionals who participated provided a representative picture of their views and practices regarding physical activity during pregnancy. Secondly, the use of a targeted questionnaire with well-defined questions ensured the coverage of the variables under investigation. Furthermore, the statistical analyses performed were extensive and detailed, providing robust findings and allowing clear conclusions to be drawn. Lastly, this study makes an important contribution to the literature, as it provides new information about the practices of healthcare professionals in Greece and identifies the factors that influence their recommendations for physical activity during pregnancy, which may lead to improvements in educational and professional practices in this field.

Despite the novelty of the study, given the lack of relevant data from Greece, it bears certain limitations that need to be addressed. An important limitation is its cross-sectional design, which limits the possibility of detecting a causal relationship between participants’ sociodemographic and occupational characteristics and their recommendations for physical activity during pregnancy [40]. A limitation of this study is that the questionnaire was developed by the research team without formal validation or reliability testing. While it was designed based on the relevant literature and expert input to align with the study’s objectives, future research should consider validating the instrument to ensure its psychometric robustness. Data were collected at a specific point in time [41], which may not capture possible changes in views and practices during different stages of healthcare professionals’ careers or changes of knowledge over time. In addition, the study relies on self-reported data, which can introduce bias due to participants’ tendency to respond in a socially desirable manner or due to recall inaccuracy [42]. The sample is also geographically limited to the Attica region, which may limit the generalizability of the findings to other regions of the country or other countries with different health systems, cultural views on physical activity, and training programs for health professionals. A key limitation of this study is the absence of a priori sample size calculation. The sample was determined based on feasibility and the willingness of healthcare professionals to participate, which may affect the generalizability of the findings. Future studies should incorporate formal sample size estimations to ensure adequate statistical power. Finally, the study did not consider other potentially influential factors, such as personal experience with pregnancy, specific training in physical activity during pregnancy, or the availability of resources to support physical activity recommendations, which could further explain the observed behaviors and practices.

## 5. Conclusions

The present study highlights the importance of recommendations from healthcare professionals, and more particularly midwives and obstetricians, for physical activity during the second and third trimesters of pregnancy. This study underscores the critical role of healthcare professionals in promoting physical activity during pregnancy and highlights the impacts of sociodemographic and professional factors on their recommendations. The findings suggest that targeted interventions should consider these factors to enhance the consistency and accuracy of exercise recommendations. Specifically, training programs for midwives and obstetricians should integrate evidence-based guidelines on antenatal physical activity, ensuring that healthcare professionals feel confident in advising pregnant women. Additionally, standardizing recommendations across different healthcare settings could improve the uniformity of guidance provided to expectant mothers. Future interventions should focus on increasing healthcare professionals’ awareness of international guidelines and fostering a more structured approach to exercise counseling during pregnancy. Moreover, policy initiatives could support the inclusion of physical activity education in medical and midwifery curricula, equipping future professionals with the necessary knowledge and skills. Further research is needed to explore long-term trends in healthcare professionals’ recommendations and assess the effectiveness of tailored educational programs in improving physical activity promotion during pregnancy.

## Figures and Tables

**Table 1:** Practices and recommendations for physical activity during the second and third trimesters of pregnancy (*n* = 235).

	Second Trimester		Third Trimester	
	*n*	*%*	*n*	*%*
**Recommend physical activity**				
No	3	1.28	5	2.13
Yes	232	98.72	230	97.87
Total	235	100.0	235	100.0
**Recommend cardio exercises**				
No	16	6.81	27	11.47
Yes	219	93.19	208	88.51
Total	235	100.0	235	100.0
**Recommend resistance/strengthening exercises**				
No	131	55.74	134	57.02
Yes	104	44.26	101	42.98
Total	235	100.0	235	100.0
**Recommend relaxation activity**				
No	29	12.34	24	10.21
Yes	206	87.66	211	89.79
Total	235	100.0	235	100.0
**Recommended frequency**				
Not recommended	55	24.0	56	23.8
1 day/week	2	0.8	3	1.3
2–3 days/week	130	54.9	121	52.0
4–5 days/week	32	13.5	22	9.3
5–6 days/week	1	0.4	8	3.4
Everyday	15	6.3	24	10.1
Total	235	100.0	234	100.0
**Duration of physical activity**				
Not recommended	50	21.9	56	24.0
15–30 min	84	35.4	71	30.9
30–45 min	83	35.0	78	32.9
>1 h	4	1.7	13	5.5
No limit	14	5.9	16	6.8
Total	235	100.0	234	100.0

**Table 2:** Occupational characteristics associated with recommendations regarding physical activity during the second trimester of pregnancy (*N* = 235).

		**Recommend Relaxation Activity During Second Trimester**			
		*n* (*%*)			
		No	Yes	*χ* ^2^	*p*
*Midwives’ experience in prenatal counseling*	No	8 (3.4)	13 (5.5)	3.849	0.050
	Yes	25 (10.6)	106 (45.1)		
		**Recommend resistance/strengthening exercises during second trimester**			
		*n (%)*			
		No	Yes	*χ* ^2^	*p*
Specialty	Obstetrician	38 (16.1)	45 (19.1)	6.613	0.010
	Midwife	96 (40.8)	56 (23.8)		
		*n* *(%)*			
Education		No	Yes	*χ* ^2^	*p*
	Tertiary education	78 (33.1)	48 (20.4)	4.457	0.035
	MSc/PhD	52 (22.1)	56 (23.8)		
		*n* *(%)*			
		No	Yes	*χ* ^2^	*p*
Work sector	Public sector	46 (19.5)	26 (11)	8.222	0.042
	Primary healthcare	25 (10.6)	13 (5.5)		
	Private sector	18 (7.6)	13 (5.5)		
	Freelance	42 (17.8)	52 (22.1)		
		*n* *(%)*			
		No	Yes	*Fisher’s Exact test*	*p*
Obstetricians’ position	Director/curator/professor	7 (2.9)	13 (5.5)	6.852	0.031
	Trainee obstetrician	12 (5.1)	5 (2.1)		
	Freelance	16 (6.8)	30 (12.7)		
		*n* *(%)*			
		No	Yes	*χ* ^2^	*p*
*Midwives’ experience in prenatal counseling*	No	27 (11.4)	69 (29.3)	6.308	0.012
	Yes	6 (2.5)	50 (21.2)		

**Table 3:** Occupational and demographic characteristics associated with recommendations regarding physical activity during the third trimester of pregnancy (*N* = 235).

		**Recommend Cardio Exercises During Third Trimester**			
		*n* *(%)*			
		No	Yes	***χ* ^2^**	***p***
*Participant’s gender*	Man	12 (5.1)	47 (20)	6.067	0.014
	Woman	15 (6.3)	161 (68.5)		
		*n* *(%)*			
*Midwives’ experience in prenatal counseling*		No	Yes	*χ* ^2^	*p*
	No	10 (4.2)	6 (2.5)	17.504	0.001
	Yes	23 (9.7)	113 (48)		
		**Recommend resistance/strengthening exercises during third trimester**			
		*n* *(%)*			
*Midwives’ experience in prenatal counseling*		No	Yes	*χ* ^2^	*p*
	No	28 (11.9)	5 (2.1)	8.823	0.004
	Yes	68 (28.9)	51 (21.7)		

**Table 4:** Occupational and demographic characteristics associated with recommendations regarding the frequency and duration of physical activity during the third trimester of pregnancy (*N* = 235).

		**Duration of Physical Activity During the Third Trimester**					
		*n* (%)					
		Not recommended	1/2–3 days/week	4–5/5–6 days/week&everyday		*χ* ^2^	*p*
*Work sector*	Public sector	26 (11)	33 (14)	13 (5.5)		16.056	0.013
	Primary healthcare	13 (5.5)	17 (7.2)	8 (3.4)			
	Private sector	6 (2.5)	17 (7.2)	8 (3.4)			
	Freelance	11 (4.6)	57 (24.2)	25 (10.6)			
		*n (%)*					
*Midwives’ experience in prenatal counseling*		Not recommended	1/2–3 days/week	4–5/5–6 days/week&Everyday		*χ* ^2^	*p*
	No	13 (14)	15 (6.3)	5 (2.1)		5.979	0.050
	Yes	24 (10.2)	59 (25.1)	35 (14.8)			
		**Frequency of resistance/strengthening exercises during third trimester**					
		*n* (%)					
*Midwives’ work sector*		Not recommended	1/2–3 days/week	4–5/5–6 days/week&everyday		*χ* ^2^	*p*
	Hospital/primary healthcare	13 (14)	15 (6.3)	5 (2.1)		6.720	0.035
	Freelance	24 (10.2)	59 (25.1)	35 (14.8)			
		**Suggested duration of physical activity during the third trimester**					
		*n* (%)					
*Gender*		Not recommended	15–30 min	30–45 min	>1 h/ No limit	*χ* ^2^	*p*
	Man	16 (6.8)	28 (11.9)	10 (4.2)	5 (2.1)	15.283	0.002
	Woman	40 (17)	43 (18.2)	68 (28.9)	24		
		*n (%)*					
		Not recommended	15–30 min	30–45 min	>1 h/ No limit	*χ* ^2^	*p*
*Specialty*	Obstetrician	23 (9.7)	36 (15.3)	16 (6.8)	8	16.378	0.001
	Midwife	33 (14)	35 (12.7)	62 (26.3)	21		

## Data Availability

All data generated or analyzed during this study are included in the article as Tables. Any other data requirement can be directed to the corresponding author upon reasonable request due to the data not being publicly available due to containing information that could compromise the privacy of the research participants.
